# Hind wing variation in *Leptura annularis* complex among European and Asiatic populations (Coleoptera, Cerambycidae)

**DOI:** 10.3897/zookeys.724.20667

**Published:** 2017-12-21

**Authors:** Robert Rossa, Jakub Goczał, Bartosz Pawliczek, Nobuo Ohbayashi

**Affiliations:** 1 Institute of Forest Ecosystem Protection, Faculty of Forestry, University of Agriculture in Krakow, 29 Listopada 46, 31-425 Krakow, Poland; 2 Kamimiyada 1334-444, M; 3 iura City, Kanagawa, 238-0101 Japan

**Keywords:** *Leptura annularis*, longhorn beetles, geometric morphometrics, geographic variation, taxonomy

## Abstract

The ability to quantify morphological variation is essential for understanding the processes of species diversification. The geometric morphometrics approach allows reliable description of variation in animals, including insects. Here, this method was used to quantify the morphological variation among European and Asiatic populations of *Leptura annularis* Fabricius, 1801 and its closely related species *L. mimica* Bates, 1884, endemic for Japan and Sakhalin islands. Since the taxonomic status of these two taxa is differently interpreted by taxonomists, they are collectively called “*Leptura annularis* complex” in this paper. The analysis was based on the measurements of hind wings of 269 specimens from six populations from Europe and Asia. The level of morphological divergence between most of continental European and Asiatic populations was relatively small and proportional to the geographic distance between them. However, distinct morphotype was detected in Sakhalin Is. and Japan. These data confirm the morphological divergence of the endemic *L. mimica* species. Obtained results highlight the potential of the geometric morphometric method in studying morphological variation in beetles.

## Introduction

The understanding of large-scale patterns of variation in living organisms is a fundamental challenge for biological science (MacArthur 1972, Gaston and Blackburn 2000). Insects have become widely used models for studying the geographical patterns of morphological variation in body size and body shape (Yom-Tov and Geffen 2006, Stillwell et al. 2007, Abbasi 2009, Sadeghi et al. 2009, Stillwell and Fox 2009). The development of rigorous method of shape analysis, the geometric morphometrics, has provided new opportunities in the morphological study on animals (Adams et al. 2004, Zelditch et al. 2004, Lawing and Polly 2010), including insects (Pezzoli et al. 1997, Haas and Tolley 1998, Hoffmann and Shirriffs 2002).

The Cerambycidae family constitutes a large and diverse group of beetles. Among them, there are species with highly limited distribution or even endemics, as well as widely distributed and common taxa (Löbl and Smetana 2010). Longhorn beetles differ also in terms of habitat specialization: from highly-specialized monophagous species to polyphagous opportunists able to inhabit various habitats. The role of ecological and historical factors on Cerambycidae distribution is relatively well studied (Baselga 2008, Koutroumpa et al. 2013, Vitali and Schmitt 2017). However, there is a lack of papers devoted to quantification of the geographical patterns in morphological variation of longhorn beetles.

*Leptura annularis* is a widely distributed longhorn beetle which taxonomic status remains unclear. In 1801, the species was described as *L. annularis* by Fabricius, based on the sample from Siberia (Fabricius 1801). In 1884, a new species, *Leptura mimica*, was described by Bates, based on specimens from Hokkaido and Honshu (Bates 1884). Nevertheless, many authors have later synonymized these two taxa (e.g., Panin and Săvulescu 1961, Kaszab 1971, Cherepanov 1988, Sláma 1998, Sama 2002) and indicated that there are no significant differences between populations from Europe, Asia, and Sakhalin Is. or Japan (Sama 2002) or treat these two taxa as subspecies but not distinct species (Danilevsky 2014).

On the other hand, comprehensive studies conducted by Japanese taxonomists have indicated significant differences between continental populations of *L. annularis* and populations of *L. mimica* distributed in Japan and Sakhalin. Such differences can be found in elytra coloration pattern, shape of male genitalia parameres and female spermatheca (Makihara and Saito 1985, Makihara et al. 1991). Moreover, the study on mitochondrial genome suggests that *L. annularis* and *L. mimica* should be considered as separate species (Saito et al. 2002). In this study, these two taxa are collectively called the “*Leptura annularis* complex”.

So far, all morphological studies on *L. annularis* complex were based on the traditional, qualitative characters only. Therefore, the main aim of this study was to quantify the morphological variation between European and Asiatic populations of *Leptura annularis* complex by using a geometric morphometric approach. This will allow examination of the hypothesis that the Sakhalin Is. and Japanese populations of the studied species constitute a diffrent morphotype than the continental populations.

## Materials and methods

### Examined material

**Figure 1.**
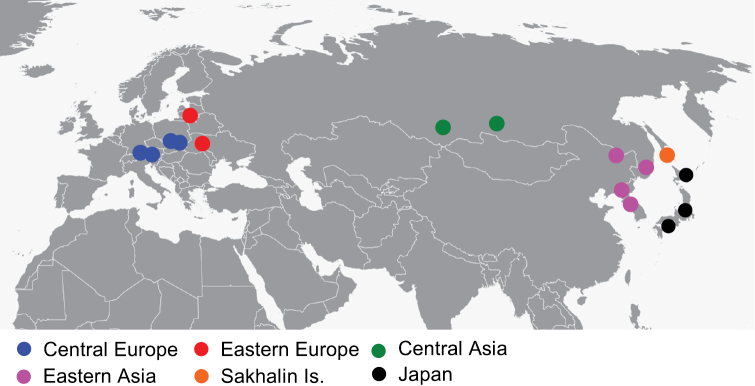
Sampling localities for morphological survey of *Leptura annularis* complex in Europe and Asia.

The study was based on analysis of 269 images (116 females, 153 males) originating from six populations (Fig. 1): Central Europe (121 specimens), Eastern Europe (28 specimens), Central Asia (13 specimens), Eastern Asia (60 specimens), Sakhalin Is. (10 specimens), and Japan (37). Specimens were obtained from museum collections at the Institute of Forest Ecosystem Protection, Faculty of Forestry, University of Agriculture in Krakow, Poland, from collections of the Nature Museum at the Institute of Systematics and Evolution of Animals of the Polish Academy of Science, Krakow, Poland and from private collection of Nobuo Ohbayashi. Specimens were collected between 1888 and 2015.

### Measurements

**Figure 2.**
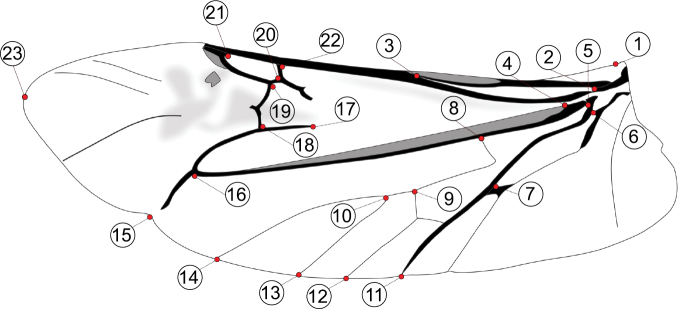
Schematic of landmarks positions on the hind wing of *Leptura annularis* complex.

Both left and right hind wings of each specimen were carefully detached from the body, straightened, and mounted between two microscopic slides (Goczał et al. 2016). Each preparation was digitalized using an Epson V330 Photo scanner with a resolution of 4,800 dpi. Subsequently, 23 homologous landmarks were determined manually on each wing image by using of DrawWing software (Tofilski 2004) (Fig. 2).

### Statistical analyses

Measurements of left and right hind wing were averaged. Before the analysis, all coordinates of the landmarks were aligned by using generalized orthogonal least-squares procedures (Rohlf and Slice 1990). These procedures involve scaling, translation and rotation of the landmarks. After the superposition, coordinates of landmarks can be compared. Wing size was expressed as a centroid size. Wing shape was described by 20 principal components. The ANOVA/MANOVA models were used to analyze the differences in hind wing size and shape between populations and sexes. Mahalanobis distance (MD) was used as a measure of morphological divergence between groups. The distances were also employed to build a similarity tree by using of Unweighted Pair Group Method with Arithmetic Mean (UPGMA) in the Phangorn package (Schliep 2011) in R software(R Core Team 2015).

## Results

### Size differences

**Figure 3.**
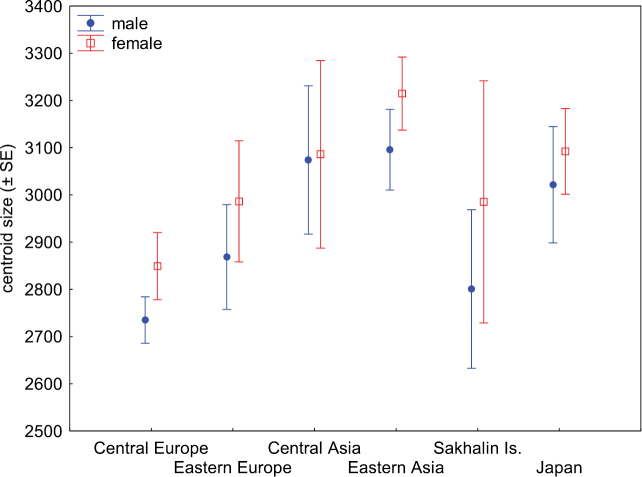
Differences in wing size between six populations of *Leptura annularis* complex.

Significant differences in average wing size were detected among populations of *L. annularis* complex (ANOVA: F_5, 257_ = 22.56, *P* = 0.001, Fig. 3) and between sexes (ANOVA: F_1, 257_ = 6.02, *P* = 0.015, Fig. 3). The interaction between population and sex was not significant (ANOVA: F_5, 257_ = 0.27, *P* = 0.931). The post-hoc test revealed that specimens from Central Asia, Eastern Asia and Japan were significantly larger than individuals from Central Europe (Scheffe Test: *P* = 0.001; *P* = 0.001; *P* = 0.001, respectively). Specimens from Eastern Asia were also smaller than individuals from Eastern Europe and Sakhalin Is. (Scheffe Test: *P* = 0.001; *P* = 0.009, respectively). Other populations did not differ significantly in hind wing size.

### Shape differences

**Figure 4.**
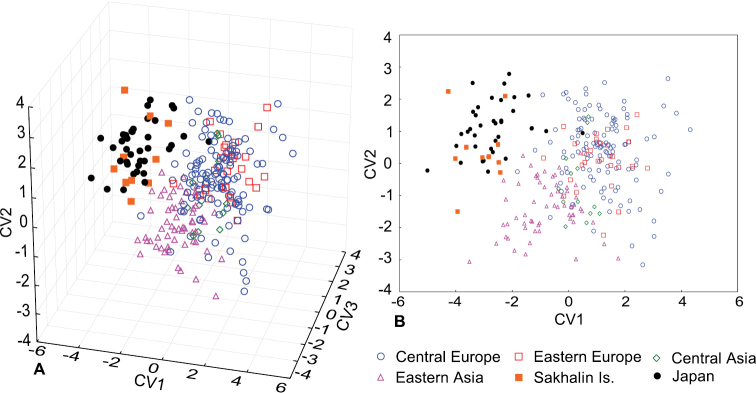
Variation of hind wing shape among European and Asiatic populations of *Leptura annularis* complex: view in three-dimensional (**A**) and two-dimensional (**B**) morphospace.

**Figure 5.**
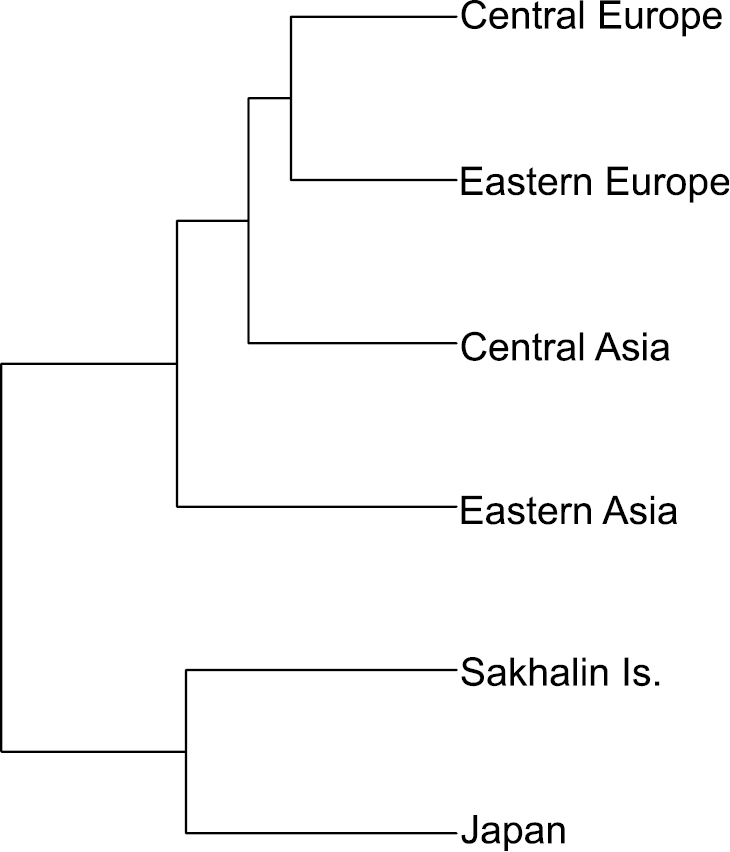
UPGMA similarity tree of hind wing shape of six *Leptura annularis* complex populations based on the Mahalanobis distance.

There were significant differences in hind wing shape among populations of *L. annularis complex* (MANOVA: Wilks’ lambda = 0.14, F_100, 1165.8_ = 5.91, *P* = 0.001, Fig. 4) and between sexes (MANOVA: Wilks’ lambda = 0.70, F_20, 238_ = 5.07, *P* = 0.001). The interaction between population and sex was not significant (MANOVA: Wilks’ lambda = 0.63, F_100, 1165.8_ = 1.14, *P* = 0.168). Morphological divergence among populations from Central Europe, Eastern Europe, Central Asia and Eastern Asia have reflected in large degree the geographical distance between them (Figs 4, 5). Accordingly, specimens from Central Europe were most similar to the individuals from Eastern Europe (MD square = 2.1). Individuals from Central Asia were similar to the specimens from Eastern Europe (MD square = 3.1). Specimens from Eastern Asia were similar to the beetles from Central Asia (MD square = 3.8).

Populations from Sakhalin Is. and Japan have shown significant divergence from all continental populations (Figs 4, 5), including the relatively close Eastern Asia population (MD square = 12.5; 9.8, respectively). Furthermore, samples from Sakhalin Is. and Japan were more similar to each other (MD square = 5.7) than to any continental population.

Discriminate analysis allowed to separate samples from Sakhalin Is. and Japan from continental populations based on hind wing shape (*P* = 0.001). Nevertheless, discrimination accuracy was relatively low and adopted values between 86.5 % (with cross-validation) for identification of continental morphotype, and 87.2 % (with cross-validation) for discrimination of Sakhalin Is. and Japanese morphotype.

**Figure 6.**
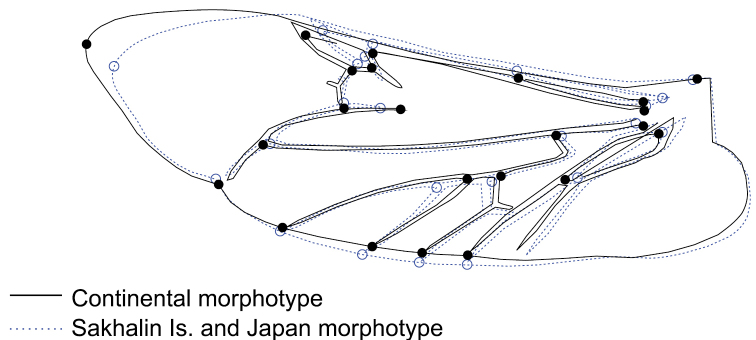
Differences in average hind wing shape between continental *Leptura annularis* complex morphotype (full line) and morphotype from Sakhalin Is. and Japan (dotted line). Differences were exaggerated four times to make them more visible. The position of the lines is a result of interpolation, which is less accurate at greater distances from the landmarks. The presented differences are difficult to discern without measurements.

The average hind wing of *L. annularis* from Sakhalin Is. and Japan was slightly shorter than the hind wing of specimens from continental populations, and has wider wing tip (Fig. 6). Differences may be also found in the position of some wing veins including cubital and medial veins (Fig. 6). However, these differences were very small and difficult to discern without measurements.

## Discussion

Significant differences in size and shape of hind wings were found among European and Asiatic populations of *Leptura annularis* complex. The level of morphological divergence between most of studied populations was relatively small and proportional to the geographic distance between them. These data suggest that the postglacial colonization of Europe and Asia by *L. annularis* probably originated from single refugium.

The only exception to this pattern was in the case of Japanese and Sakhalin Is. populations. Samples from this region constituted a distinct morphotype, and differences between them and continental populations cannot be explained simply by the geographical distance. These data correspond to the results of other morphological and genetic investigations which have shown clear morphological divergence of Japan and Sakhalin Is. populations (Makihara and Saito 1985; Saito et al. 2002) and confirm the validity of taxonomic status of endemic *L. mimica* species.

The development of the geometric morphometric method is considered to be a milestone in the field of morphological study (Rohlf and Marcus 1993). Replacement of simple linear measurements with the complex informations of shape allows examination of various taxonomic, ecological, and evolutionary hypotheses (Adams et al. 2004, Mitteroecker and Gunz 2009, Lawing and Polly 2010, Fruciano 2016). In the case of insects, flight wings with their relatively flat area and numerous homologous structures constitute a widely used marker in geometric morphometric investigations (e.g. Bai et al. 2012; Chazot et al. 2016; Francoy et al. 2011; Gilchrist et al. 2000; Perrard et al. 2014; Prudhomme et al. 2012; Sadeghi et al. 2009; Tofilski 2008). In the case of beetles, hind wing geometric morphometrics were successfully used to describe the geographical variation among populations (Mikac et al. 2016, Rossa et al. 2016) and for species identification (Su et al. 2015, Goczał et al. 2016, Li et al. 2016, Rossa et al. 2017), as well as in evolutionary investigations (Bai et al. 2012; Ren et al. 2017). The results presented here confirmed that this approach is suitable for describing the geographic pattern of morphological variation in longhorn beetles and allows detection of divergent morphotypes. These findings highlight the potential of the geometric morphometric method in studying morphological variation in Coleoptera.

It is well known that habitat specialization constitutes an important factor affecting distribution patterns and diversification of organisms (Caillaud 1999, Wood et al. 1999, Stireman et al. 2005). In general, opportunistic species are in many cases characterised by a more homogeneous population structure than highly specialized taxa (Smith and Fujio 1982, Mustaparta 1992, Stein et al. 2014). If the case of longhorn beetles, it was shown that host specialization was an important factor influencing the distribution patterns and diversification of this group (Shoda et al. 2003b, Vitali and Schmitt 2017, Wallin et al. 2017). Our investigation on *L. annularis* showed a homogenous morphological structure of the studied species over a large distribution range. A similar conclusion was drawn for the other opportunistic longhorn beetle *Anoplophora glabripennis* (Motschulsky, 1853) after the genetic investigation (Carter et al. 2009). In contrary, several studies on host-specific longhorn beetles revealed more complex morphological and genetic population structures that cannot be explained simply by the geographical distance (Shoda et al. 2003a, 2003b, Kawai et al. 2006, Rossa et al. 2016). These findings underscore the importance of host specialization in the distribution patterns and diversification of longhorn beetles.
